# Development of a Microfluidic Point-of-Care Platform for HPV Detection Based on Helicase-Dependent Amplification

**DOI:** 10.3390/tropicalmed10090272

**Published:** 2025-09-19

**Authors:** Everardo González-González, Elda A. Flores-Contreras, Gerardo de Jesús Trujillo-Rodríguez, Mariana Lizbeth Jiménez-Martínez, Iram P. Rodríguez-Sánchez, Adriana Ancer-Arellano, Salomon Alvarez-Cuevas, Margarita L. Martinez-Fierro, Iván A. Marino-Martínez, Idalia Garza-Veloz

**Affiliations:** 1 Molecular Medicine Laboratory, Unidad Académica de Medicina Humana y Ciencias de la Salud, Universidad Autónoma de Zacatecas, Zacatecas 98160, Mexico; dnarnaprot@gmail.com (E.G.-G.); entogerry36@gmail.com (G.d.J.T.-R.); margaritamf@uaz.edu.mx (M.L.M.-F.); 2 Departamento de Patología, Facultad de Medicina, Universidad Autónoma de Nuevo León, Francisco I. Madero y Dr. E. Aguirre Pequeño s/n, Mitras Centro, Monterrey 64460, Mexico; elda.florescn@uanl.edu.mx (E.A.F.-C.); adar7035@gmail.com (A.A.-A.); salomon.alvarezcv@uanl.edu.mx (S.A.-C.); 3 Laboratorio de Fisiología Molecular y Estructural, Facultad de Ciencias Biológicas, Universidad Autónoma de Nuevo León, San Nicolás de los Garza 66455, Mexico; mariana.jimenez80@gmail.com (M.L.J.-M.); iram.rodriguezsa@uanl.edu.mx (I.P.R.-S.)

**Keywords:** HPV, diagnostics, HDA, microfluidics, POC, PCR, isothermal amplification

## Abstract

Human papillomavirus (HPV) is the most prevalent sexually transmitted infection worldwide and a leading cause of cervical cancer, accounting for over 300,000 deaths annually, primarily due to high-risk genotypes HPV-16 and HPV-18. Conventional molecular diagnostic methods, such as polymerase chain reaction (PCR), require expensive instrumentation and well-equipped laboratories, which limits their applicability in low-resource or decentralized settings. To address this challenge, the aim of this study was to develop a prototype point-of-care (POC) diagnostic platform based on helicase-dependent amplification (HDA) integrated into a microfluidic device for the specific detection of HPV-16 and HPV-18. The proposed POC platform comprises a disposable poly (methyl methacrylate) (PMMA) microfluidic device, a portable warming mat for isothermal amplification at 65 °C, and a compact electrophoresis chamber for fluorescence-based visualization using SYBR Safe dye, with an approximate total cost of $320 USD. Platform validation was performed on 33 samples, demonstrating amplification of target sequences in less than 60 min with only 20 µL of reaction volume, a limit of detection (LOD) of 15 copies (cp) per reaction, a sensitivity of 95.52%, and a specificity of 100%. This portable and scalable platform constitutes a cost-effective and reliable tool for the detection of HPV, supporting global health initiatives, including those driven by the World Health Organization (WHO), aimed at eliminating cervical cancer as a public health threat, as it can be implemented in decentralized or resource-limited settings.

## 1. Introduction

The human papillomavirus (HPV) is a non-enveloped, double-stranded DNA virus with a circular genome, classified within the *Papillomaviridae* family [1]. It is primarily spread through skin-to-skin contact and represents the most common sexually transmitted infection globally. HPV predominantly targets the reproductive tract by infecting the epithelial cells of the skin and mucous membranes [2,3].

HPV is one of the leading causes of cervical cancer in women, accounting for approximately 660,000 new cases and 350,000 deaths worldwide in 2022 [4].

Given its high prevalence and significant impact on global health, the World Health Organization (WHO) has launched the “90-70-90” Global Strategy to Eliminate Cervical Cancer, aiming to achieve elimination by 2030 [4]. The strategy includes three key targets:

1. Vaccinate 90% of girls under 15 years of age.
2. Ensure that 70% of women receive high-throughput screening in two rounds: the first before age 35 and the second before age 45.
3. Provide appropriate treatment for 90% of women diagnosed with HPV infection, precancerous lesions, or invasive cervical cancer.

To support the second and third pillars of this strategy, it is essential to implement accessible, accurate, efficient diagnostic and monitoring tools that provide rapid results. Current molecular diagnostic tests for HPV primarily target high-risk genotypes associated with the development of cervical cancer, including types 16 and 18. These genotypes are responsible for over 70% of cervical cancer cases. Detection is commonly carried out using end-point or quantitative PCR (qPCR) techniques [4,5,6]. Although these tests offer high sensitivity and specificity, they require a specialized infrastructure, highly trained personnel, and expensive equipment, such as thermal cyclers [7]. Consequently, the establishment of a molecular diagnostic laboratory can exceed US $100,000, with total cost varying depending on infrastructure, equipment, and operational scales, thus limiting their viability in low-resource settings [8].

To overcome these limitations, point-of-care (POC) diagnostic tests have emerged as viable alternatives, such as isothermal amplification assays. These tests operate at a constant temperature and enable the amplification of specific DNA sequences, delivering results in less than 60 min [9,10,11,12]. Their simplicity allows testing to be performed near or at the location of the patient, significantly reducing reliance on complex laboratory infrastructure [13]. Moreover, they offer the added advantage of enabling accessible, preventive, and timely diagnoses [9,10,11].

Helicase-dependent amplification (HDA) is one of the notable isothermal amplification methods, which utilizes a DNA helicase (UvrD from *Escherichia coli*), single-stranded DNA-binding proteins (SSB), and two specific primers to amplify the target sequence [14]. Helicase separates the duplex DNA strands, and SSB proteins prevent strand reannealing, allowing the primers to bind to the template strands. The polymerase synthesizes the complementary strand from the primers [14,15]. In contrast to loop-mediated isothermal amplification (LAMP), the HDA test employs a simpler primer design similar to conventional PCR, requiring no more than two primers and no complex secondary structures. Notably, LAMP exhibits a higher false-positive rate (up to ~28%), mainly due to nonspecific amplification of non-target DNA, such as primer dimers and contaminating DNA, while HDA exhibits a considerably lower rate (<10%) [14,16,17,18,19]. This simplicity makes HDA a more straightforward alternative among isothermal amplification techniques. Isothermal methods are usually combined with microfluidic techniques and biosensors and which allow for a lower detection limit and reaction volume [9,20,21,22]. To date, this combination has primarily involved LAMP with microfluidic chips for the detection of high-risk HPV genotypes [23,24,25]. Although HDA has been successfully applied for detecting bacteria such as *E. coli*, methicillin-resistant *Staphylococcus* strains, *Listeria monocytogenes*, hepatitis B virus, coronavirus, and monkeypox virus, no microfluidic HDA platform has yet been developed for HPV detection [26,27,28,29,30,31].

In this context, the aim of this study was to develop a POC diagnostic platform based on HDA, integrated into a microfluidic device, for the specific detection of HPV genotypes 16 and 18.

## 2. Materials and Methods

### 2.1. Ethical Considerations

Participants selected for the study were all over 18 years of age. Individuals with mental disabilities and allergies to the medications used in the treatment were excluded. Written informed consent was obtained from all patients for the collection, storage, and subsequent analysis of their samples. Sample analysis was performed anonymously to ensure participant confidentiality. The study protocol was reviewed and approved by the Ethics Committee of Hospital Universitario “Dr. José Eleuterio González”, Universidad Autónoma de Nuevo León (Monterrey, Nuevo León, Mexico) under protocol number PA15-001.

### 2.2. Sample Collection and Processing

Thirty cervical histological tissue samples from Mexican patients were collected between 2024 and 2025 at the Hospital Universitario “Dr. José Eleuterio González”. Genomic DNA was extracted from 25 mg of tissue using the NucleoSpin^®^ Tissue Kit (Macherey-Nagel, Düren, Germany), following the manufacturer’s protocol. DNA concentration and purity were assessed using a NanoDrop 1000 spectrophotometer (Thermo Fisher Scientific, Waltham, MA, USA). Purity was evaluated based on the absorbance ratios at 260/280 and 260/230 nm. DNA concentrations in the samples ranged from 10 to 100 ng/µL. Extracted DNA samples were stored at −20 °C until further analysis.

### 2.3. Genotyping of DNA Samples

The collected DNA samples were analyzed using the QuantStudio™ 3 real-time PCR system (Thermo Fisher Scientific, Waltham, MA, USA), employing the GeneProof HPV PCR kit (GeneProof, Brno-jih, Czech Republic). This kit allows the detection of high-risk HPV genotypes 16, 18 and 45 through genotype-specific fluorescent probes (Table 1).

In addition, three controls were included: one sample positive for low-risk HPV genotype 6 (sample 31, Table 1) and another sample positive for *Chlamydia trachomatis* but negative for HPV (sample 32, Table 1). Both were kindly provided by the Department of Pathology at the Universidad Autónoma de Nuevo León. The third control was a plasmid containing the Porcine Reproductive and Respiratory Syndrome virus (PRRSV) sequence, synthesized by GenScript (Piscataway, NJ, USA) (sample 33, Table 1); the plasmid map and integrated viral sequence are provided in the Supplementary Materials (Figure S1).

### 2.4. Standardization of the HDA Reaction Conditions

The HDA was standardized to determine the optimal temperature and incubation time. A DNA pool (mixture of 20 samples) positive for HPV genotypes 16 and 18, extracted from cervical tissue samples, was used as a positive control. This DNA was kindly provided by the Department of Pathology at the Universidad Autónoma de Nuevo León. Regarding the negative control, nuclease-free water was included instead of a DNA template to validate the absence of contamination or the obtaining of non-specific amplification products.

The IsoAmp^®^ II Universal tHDA kit (New England Biolabs, Ipswich, MA, USA) was used to perform the HDA test. Each reaction was prepared in a final volume of 50 µL in microtubes, containing 5 µL of 10X Annealing Buffer II, 2 µL of MgSO_4_ (100 mM), 4 µL of NaCl (500 mM), 1.5 µL each of forward and reverse primers (1 µM) (Table 2), 3.5 µL of IsoAmp^®^ dNTP Solution, 3.5 µL of IsoAmp^®^ Enzyme Mix, 5 µL of DNA template (10 ng), and nuclease-free water to adjust the final volume.

Reactions were incubated at temperatures ranging from 55 °C to 65 °C for 30 to 120 min using a SimpliAmp thermal cycler (Thermo Fisher Scientific, Waltham, MA, USA). Amplification products were analyzed by electrophoresis on a 4% agarose gel stained with SYBR Safe (1×) (Thermo Fisher Scientific, Waltham, MA, USA), employing a DNA ladder from Promega (Sunnyvale, CA, USA) covering a size range of 100 to 1500 base pairs (bp). Additionally, the detection limit (LOD) was determined using a synthetic control corresponding to a purified plasmid containing sequences from HPV genotypes 16 and 18, provided by the Department of Pathology at the Universidad Autónoma de Nuevo León. The plasmid concentration was measured using a NanoDrop 1000 spectrophotometer, and the copy number per microliter was calculated using the following formula: viral copy number (cp/μL) = (6.02 × 10^−2^) × (measured value × 10^−9^)/(DNA length × 660). The plasmid was subsequently diluted to obtain a copy number range of 30 to 1, in order to determine the LOD of the HDA assay under previously standardized conditions. The resulting products were evaluated under the same electrophoretic conditions. All reactions were carried out in triplicate.

### 2.5. Design and Fabrication of the Microfluidic Device

**Figure 1:**
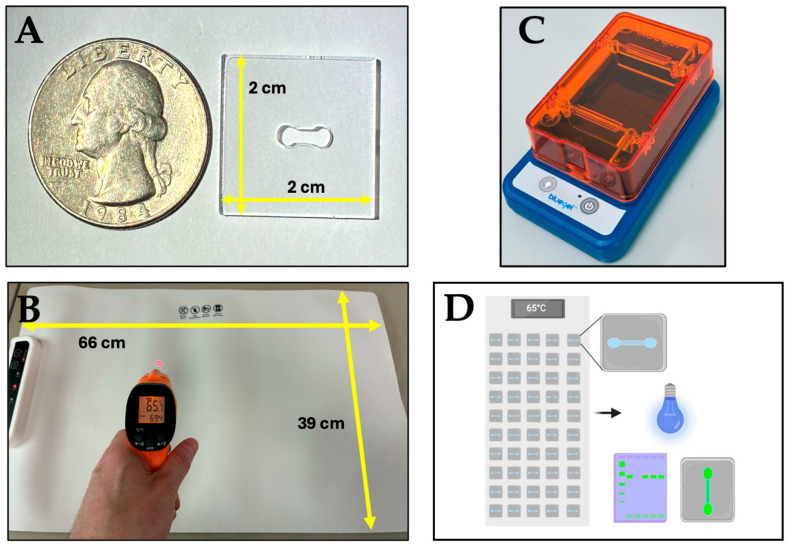
POC Platform components. (**A**) Microfluidic device. (**B**) Portable Warming Mat with its respective measurements. (**C**) Portable electrophoresis chamber. (**D**) Comprehensive schematic representation of the POC platform.

The design was created using AutoCAD 2024 (Autodesk Inc., San Francisco, CA, USA) and manufactured using poly (methyl methacrylate) (PMMA); the components were laser-cut, assembled, and sealed with chloroform. The device measures 2 cm × 2 cm, as illustrated in Figure 1A, and has two chambers with a total capacity of 20 µL, allowing for even heat distribution and leak-proofing to carry out the HDA reaction. Figure S2 shows the AutoCAD model of the microfluidic device.

### 2.6. Performance and Analysis of HDA Assays on the Microfluidic Platform

The HDA reaction integrated into the microfluidic platform was performed in a total volume of 20 µL, consisting of the following components: 2 µL of 10X Hybridization Buffer II, 0.8 µL of MgSO_4_ (100 mM), 1.6 µL of NaCl (500 mM), 0.6 µL of forward and reverse primers (1 µM) (Table 2), 1.4 µL of IsoAmp^®^ dNTP solution, 1.4 µL of IsoAmp^®^ enzyme mix, 2 µL of template DNA (1–10 ng), and nuclease-free water to complete the final volume. The reaction was performed under pre-standardized conditions, and the assay functionality was validated using 33 pre-genotyped samples. After sample loading, the microfluidic chips were incubated on a portable warming mat (DAMET, Shenzhen China) (Figure 1B) at 65 °C for 60 min. Following incubation, 1 µL of SYBR Safe (2000X stock solution) was added to each chip (33 in total), resulting in a final concentration of 20X. The chips were placed in a portable electrophoresis chamber (Amplyus LLC, Boston, MA, USA) equipped with a blue light source (Figure 1C), enabling fluorescence visualization to be correlated with the presence of HPV genotypes 16 or 18 in the samples. Furthermore, chip images were processed using ImageJ 1.53t software (National Institutes of Health, Bethesda, MD, USA), analyzing the signal intensity with the blue channel. (Figure S3). To confirm the results, electrophoresis was performed using 4% agarose gels using the portable electrophoresis chamber. Figure 1D shows the general scheme followed to perform the HDA test integrated into the microfluidic platform, and Table 3 shows the costs of the components.

### 2.7. POC Platform

The following elements were employed to carry out HPV amplification via HDA: (A) a microfluidic device, (B) a portable warming mat, and (C) a portable electrophoresis chamber. Figure 1 shows the components of the POC platform. The platform is composed of accessible and portable components, allowing the system to have a low cost (approximately $320 USD). Detection is achieved through the emission of green fluorescence in the presence of target DNA. This signal results from the binding of SYBR Safe dye to the double-stranded DNA amplified by HDA; once bound, the dye fluoresces upon excitation with blue light. The platform supports low-volume reactions (20 µL), enabling rapid detection (in less than 60 min), assessment of treatment efficacy, and monitoring of disease progression associated with HPV-related cervical cancer.

### 2.8. Statistical Analysis

Statistical analysis was performed using Minitab^®^ 2025 software (Minitab Inc., Centre County, PA, USA) using attribute agreement analysis to assess the reliability of the HDA test.

## 3. Results

### 3.1. Standardization of Reaction Conditions

**Figure 2:**
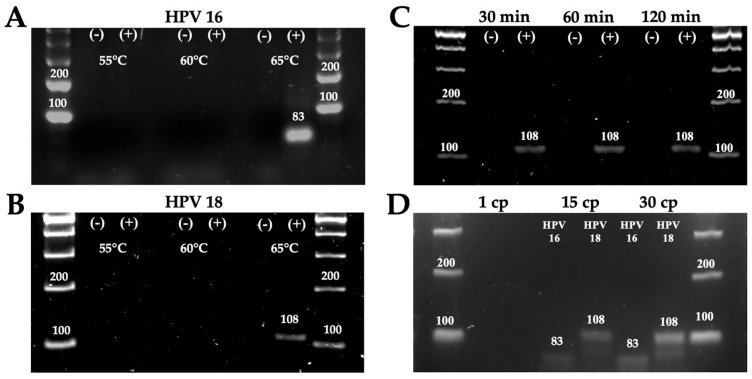
Standardization of HDA conditions. (**A**,**B**). Evaluation of incubation temperatures ranging from 55 °C to 65 °C to determine the optimal conditions for HDA of HPV genotypes 16 (**A**) and 18 (**B**). (**C**) Assessment of incubation times using HPV 18-specific primers to identify the minimum time required for detectable amplification. (**D**) Determination of the LOD of the HDA assay, using a copy number (cp) range of 30 to 1, for HPV genotypes 16 and 18.

To optimize the HDA assay for the detection of HPV genotypes 16 and 18, a temperature gradient from 55 °C to 65 °C was evaluated. A pooled DNA sample positive for HPV genotypes 16 and 18, obtained from cervical tissue, was used as the positive control. For the negative control, the amplification reaction was performed in the absence of template DNA. Reactions were prepared in 50 µL volumes using Eppendorf tubes and carried out according to the IsoAmp^®^ II Universal tHDA Kit protocol. Each reaction was incubated for 90 min. The results indicated that the optimal amplification temperature was 65 °C (Figure 2A,B). At this temperature, an amplification band of approximately 83 bp was observed for genotype 16 (Figure 2A) and a band of approximately 108 bp for genotype 18 (Figure 2B).

To establish the optimal reaction time, HDA reactions with primers targeting HPV18 were incubated for 30–120 min. Amplification products were detectable within 30 min (Figure 2C). The LOD of the assay was also evaluated using a synthetic control containing HPV16 and HPV18 sequences, under standardized conditions. The LOD was 15 cp per reaction for both genotypes (Figure 2D).

### 3.2. Detection of HPV Genotypes 16 and 18 Using the POC Platform

**Figure 3:**
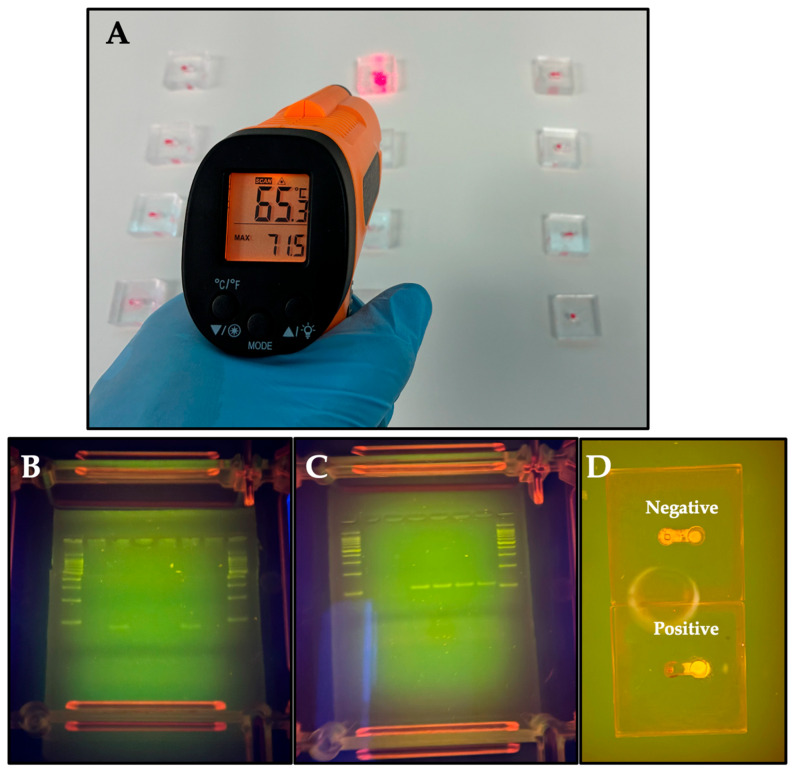
Working POC Platform for HPV detection. (**A**) A set of microfluidic devices was incubated at 65 °C utilizing a portable warming mat for temperature control. (**B**,**C**) Gel electrophoresis results showing positive bands for genotypes 16 (**B**) and 18 (**C**). (**D**) Fluorescence visualization of samples stained with SYBR Safe was performed on the microfluidic device using the electrophoresis chamber.

Following the optimization of reaction conditions in microtubes, HDA was implemented on the 2 cm × 2 cm microfluidic device (Figure 1A) using 20 µL reaction volumes. A total of thirty-three samples were processed on the chip using specific primers for HPV 16 and 18, followed by incubation at 65 °C (verified with an infrared thermometer) for 60 min on a portable warming mat (Figure 3A). A run time of 60 min was chosen due to the high variability in patient sample quality. This was done to ensure sufficient time for HDA. Post-incubation, amplification products were analyzed by electrophoresis on a 4% agarose gel and visualized under blue light using the electrophoresis chamber (Figure 1C).

In parallel, the samples were stained with SYBR Safe to enable direct, on-chip visual detection of HPV genotypes 16 and 18. Under blue light, positive samples exhibited a green fluorescent signal, while negative samples displayed an orange signal.

The results obtained by gel electrophoresis (Figure 3B,C) and SYBR Safe (Figure 3D and Figure S4) were consistent with the real-time PCR analysis (Table 1), except for sample 20, which was negative in the HDA assay and positive by real-time PCR (Table 1) and (Figure S4). Thus, the assay showed a sensitivity of 95.52% and a specificity of 100%, with a kappa of 0.936 (95% CI: 84.24–99.92, *p* < 0.05) (Table 4). Furthermore, it is important to highlight that no cross-reactions were observed between samples positive for HPV 6 (Figure S4), *Chlamydia trachomatis* (Figure S4) and PRRSV (Figure S4) when primers for the detection of HPV 16 and 18 were used. However, larger-scale studies are needed to validate these performance metrics and determine their consistency in larger clinical settings.

## 4. Discussion

Here, we report a prototype microfluidic platform integrating helicase-dependent amplification (HDA) for the simple, cost-effective detection of high-risk HPV genotypes 16 and 18 in a point-of-care (POC) setting.

Test conditions were standardized using a pooled sample containing both genotypes, enabling biological heterogeneity and the inclusion of a broader range of variables. This strategy reduces operational costs and minimizes the number of individual tests required, while ensuring reliable platform performance [32,33].

Among the findings of this POC platform is the obtaining of results around 60 min, with a LOD of 15 cp per reaction. Moreover, the platform demonstrated a sensitivity of 95.52% and a specificity of 100%, with only one false negative detected, likely due to target DNA degradation or the presence of inhibitors in the sample that interfered with isothermal amplification [34]. In addition, no cross-reactivity was observed with other HPV genotypes (e.g., type 6), sexually transmitted infections such as *Chlamydia trachomatis*, or viruses from other species such as PRRSV. These features are comparable to those reported for other isothermal amplification methods used for HPV detection, which typically require 60 to 120 min and detect target sequences using DNA inputs below 100 cp per reaction, with sensitivities and specificities ranging from 84% to 100% [9,24,35,36].

Furthermore, this microfluidic platform allows the use of a significantly reduced reaction volume of 20 µL, unlike standard HDA assays that require reaction volumes of around 50 µL, and other isothermal POC systems that often exceed reaction volumes of 30 µL, thereby reducing the overall cost of reactions [15,37,38,39]. This is possible due to the miniaturized design of the chambers, which are noticeably smaller than conventional PCR tubes (capacity of 200 µL). The reduced scale increases the efficiency of the amplification process by facilitating heat transfer, and this configuration also helps minimize reagent consumption. Another key advantage is that using a microfluidic chip instead of conventional microtubes offers significant biosafety benefits. The design reduces the risk of sample leakage, aerosol formation, and cross-contamination during amplification, common problems associated with manual handling and opening of microtubes [40]. It also provides greater protection for users and minimizes environmental exposure. Therefore, the platform’s design is ideal for deployment in low-resource settings, especially where the presence of multiple pathogens increases the risk of cross-contamination.

The isothermal nature of the HDA enabled incubation using a portable warming mat, contributing to a user-friendly and cost-efficient setup. This simple POC platform allows for the incubation of more than 60 samples per hour, resulting in a processing capacity of several hundred samples per day. Notably, the performance of this platform is comparable to that of HPV detection systems currently recommended by the Pan American Health Organization (PAHO) WHO, which offer processing capacities ranging from 45 to 471 samples per day. Nevertheless, comparative studies using clinical samples are necessary to accurately evaluate its performance and operational feasibility [41]. The flexibility of this technology allows it to be adapted to detect a wide range of pathogens, enabling large-scale diagnostics. This versatility is advantageous during public health crises, such as pandemics, where rapid and decentralized testing is essential [42,43].

On the other hand, the use of SYBR Safe as a fluorescent marker enables rapid and straightforward interpretation of results. While the application of SYBR Safe in HDA assays has not been previously reported, a study by Yamket et al. (2023) demonstrated the use of SYBR Green following HDA to detect bacterial DNA in platelet samples [38]. In that study, green fluorescence was observed under UV light in the presence of amplified products, supporting the feasibility of using intercalating dyes for post-amplification detection [38].

In this context, the platform stands out for its operational simplicity, without the need for multiple steps that complicate its use. Although there are innovative isothermal diagnostic systems, their implementation often involves extra procedural steps, for example, the system developed by Kundrod et al. (2023), which detects HPV genotypes 16 and 18 by isothermal recombinase polymerase amplification (RPA) followed by detection using lateral flow strips [44]. Similarly, the platform developed by Zamani et al. (2021) involves a more elaborate workflow, combining LAMP with CRISPR-Cas technology and a biosensor capable of detecting electrochemical signals produced by activated Cas enzymes in the presence of the amplified target DNA [45].

**Figure 4:**
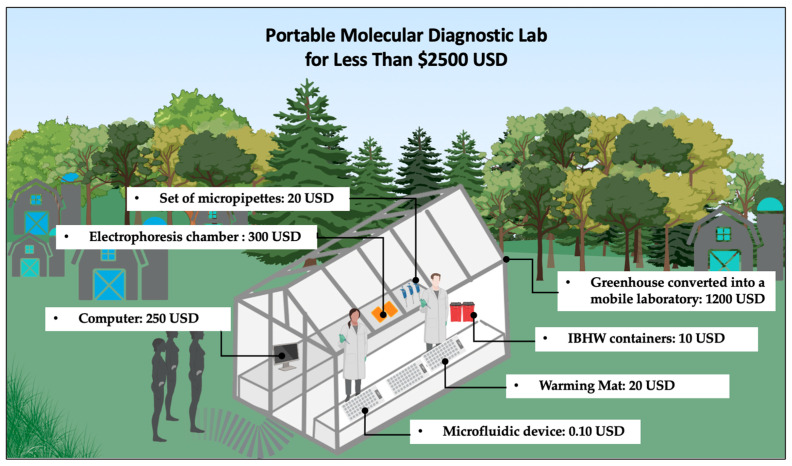
Schematic of a fully functional mobile molecular laboratory designed for use in remote areas, highlighting its main components and their estimated costs, with a total cost of less than $2500 USD.

Another significant advantage of the developed platform is its low implementation cost, estimated at US $320.10 (Table 2), which is lower than that of currently commercially available POC tests such as careHPV (Qiagen, Hilden, Germany), with instrumentation costs of around US $20,000, and GeneXpert (Cepheid, Sunnyvale, CA, USA), whose implementation costs range from US $11,530 to US $71,500 [8]. In addition, it is notably more affordable than conventional HPV diagnostic systems, which typically require equipment such as thermal cyclers and specialized laboratory infrastructure, with acquisition costs ranging from US $11,500 to US $150,000 [8,46,47]. Therefore, this platform is suitable for future implementation in low-cost mobile laboratories (at a cost of less than $2500 USD), for example, using greenhouses equipped with basic instrumentation and powered by a portable battery-based supply (Figure 4 illustrates the concept described above). This approach enables deployment in remote areas where access to molecular diagnostics is limited or unavailable. Such laboratory concepts can be of significant value in resource-limited regions and, in the context of pandemics or outbreaks, may play a critical role in controlling disease transmission and saving millions of lives.

Despite the multiple advantages demonstrated by this proof of concept, several areas of opportunity remain to be addressed. A key limitation is the need to increase the sample size for large-scale clinical validation of the platform, which is essential to establish its robustness and generalizability.

In addition, isothermal amplification on the developed platform currently requires a prior DNA extraction step. To address this, the future integration of a rapid and user-friendly DNA lysis and extraction step directly onto the chip is planned. Likewise, additional work is required to standardize and evaluate alternative fluorescent or colorimetric dyes to identify the most suitable option that allows for real-time, naked-eye interpretation of results without the need for an electrophoresis chamber, thereby improving usability in field settings. Another option is the integration of an automated DNA extraction and signal reading system, which would reduce user dependency and increase the overall reliability of the platform.

## 5. Conclusions

In this pilot study, we developed a POC platform based on isothermal HDA testing for the detection of HPV genotypes 16 and 18, achieving amplification of target DNA in 60 min, with a LOD of 15 cp per reaction. This work demonstrates that accessible, simple, and low-cost components can enable the development of practical and attractive diagnostic solutions. As a proof of concept, this platform represents a promising tool for future implementation in remote areas, where access to molecular diagnostics is often limited by high costs and infrastructure requirements. This platform costs approximately $320 USD, making it an ideal diagnostic test for the development of mobile laboratories using greenhouses and basic instrumentation. This approach will allow the establishment of functional molecular testing workspaces at a total cost of less than $2500 USD, with substantial test processing capacity, and can be applied to detect different pathogens.

## Figures and Tables

**Table 1:** Real-time PCR genotyping results.

Samples	Results/Genotype		
	16	18	45
1	−	−	−
2	+	−	−
3	−	−	−
4	−	−	−
5	+	−	−
6	−	−	−
7	−	−	−
8	−	−	−
9	−	+	−
10	−	+	−
11	−	+	−
12	−	+	−
13	+	−	−
14	+	−	−
15	−	+	−
16	−	+	−
17	+	−	−
18	−	+	−
19	−	−	−
20	+	−	−
21	−	+	−
22	+	−	−
23	−	−	−
24	+	−	−
25	−	+	−
26	+	−	−
27	−	+	−
28	−	+	−
29	+	−	−
30	−	−	−
31	−	−	−
32	−	−	−
33	−	−	−

(+): Genotype detected; (−): Genotype not detected.

**Table 2:** Sequence of primers used for the HDA assay.

Name	Sequence	Size Amplicon	Reference
Forward HPV-16	AAGCAGAACCGGACAGAGCCCA	83 bp	[15]
Reverse HPV-16	GCTTTGTACGCACAACCGAAGCG		
Forward HPV-18	ACCAGCCCGACGAGCCGAACC	108 bp	[15]
Reverse HPV-18	GCTCGAAGGTCGTCTGCTGAGCTTT		

bp: base pairs.

**Table 3:** Cost of the components of the POC platform.

Component	Cost (USD)
Microfluidic device (PMMA)	0.10
Portable warming mat	20
Electrophoresis chamber	300
**Total**	**320.10**

The total cost of the components required to perform the HDA assay integrated into the microfluidic platform is highlighted in bold.

**Table 4:** Determination of the sensitivity and specificity of the HDA assay.

HDA Assay	Real-Time PCR		Total	Sensitivity	Specificity	Kappa (95% CI, *p*-Value)
	+	−				
+	20	0	20	95.52%	100%	0.936 (84.24–99.92, *p* < 0.05)
−	1	12	13			
Total	21	12	33			

CI: Confidence interval.

## Data Availability

The original contributions presented in this study are included in the article/Supplementary Material. Further inquiries can be directed to the corresponding authors.

## Supplementary Materials

The following supporting information can be downloaded at: https://www.mdpi.com/article/10.3390/tropicalmed10090272/s1; Figure S1: DNA vector map containing viral sequences of the PRRSV, used as a negative control for the HDA assay analysis; Figure S2: AutoCAD model used for the design and fabrication of the microfluidic system; Figure S3: Image analysis of the chip with HDA reactions was generated using ImageJ1.53t software, processing data specifically from the blue channel; Figure S4: Results of agarose gel electrophoresis from the HDA assay. (A–C) Results of samples 13 to 30. (D) Results of negative controls, including HPV-6, *Chlamydia trachomatis* (CT), and PRRSV.

## Abbreviations

The following abbreviations are used in this manuscript:

bp	Base Pairs
cp	Copies
CI	Confidence interval
HDA	Helicase-Dependent Amplification
HPV	Human Papillomavirus
LOD	Limit of detection
PAHO	Pan American Health Organization
POC	Point-of-care
PMMA	Poly(methyl methacrylate) PMMA
RPA	Recombinase polymerase amplification
SSB	Single-Stranded DNA Binding Proteins
WHO	World Health Organization
