# Supplementary material for: Development of a Microfluidic Point-of-Care Platform for HPV Detection Based on Helicase-Dependent Amplification
Source: Trop Med Infect Dis. 2025 Sep 19;10(9):272. doi: 10.3390/tropicalmed10090272 (PMC12474266; doi:10.3390/tropicalmed10090272)
Supplement: Supplementary file 1 [file tropicalmed-10-00272-s001.zip › tropicalmed-3838411-supplementary.pdf]

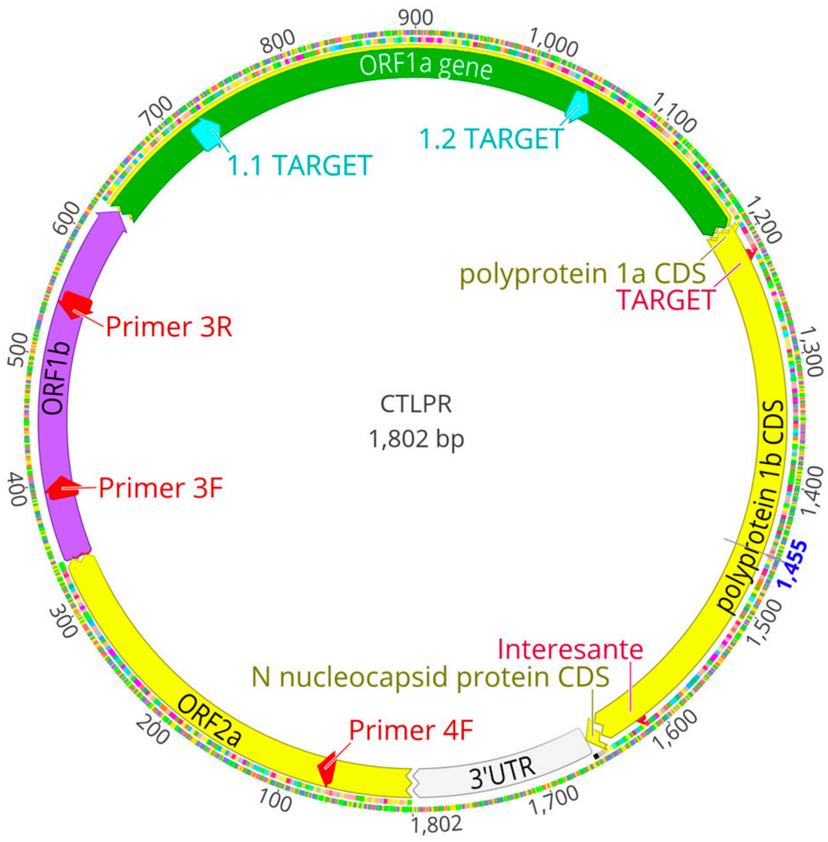

Figure S1. DNA vector map containing viral sequences of the PRRSV virus, used as a negative control for the HDA assay analysis.

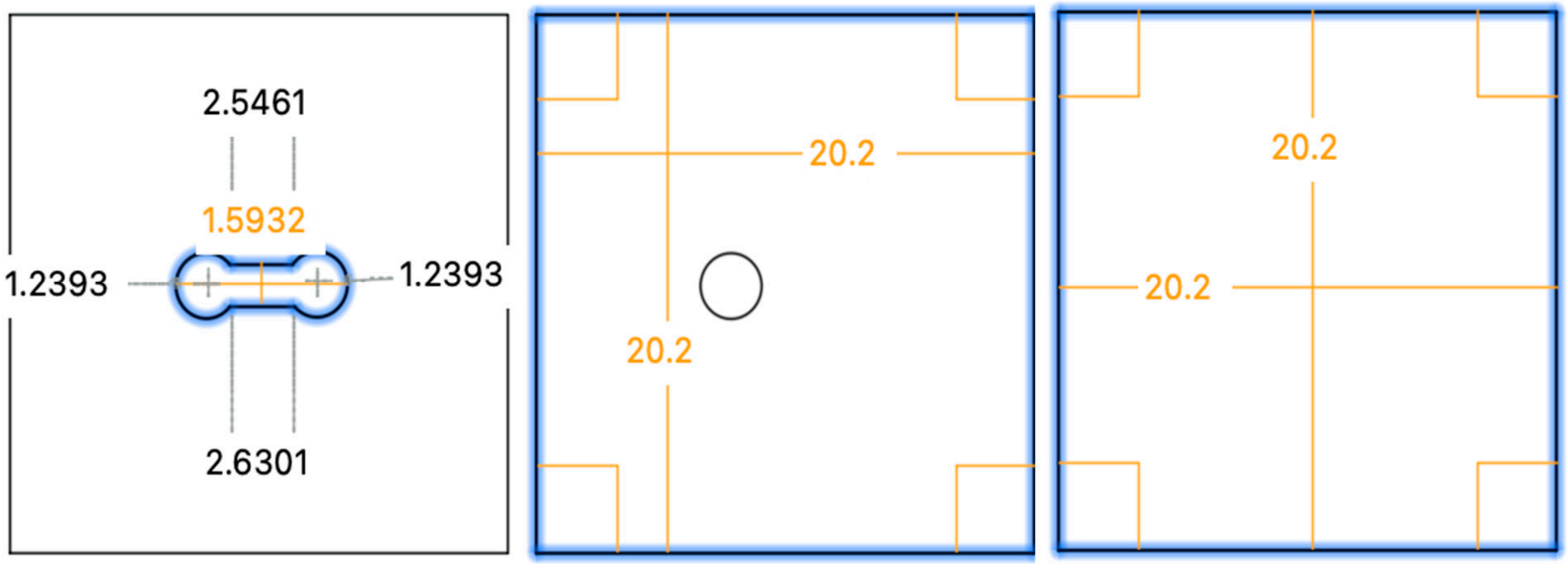

Figure S2: AutoCAD model used for the design and fabrication of the microfluidic system

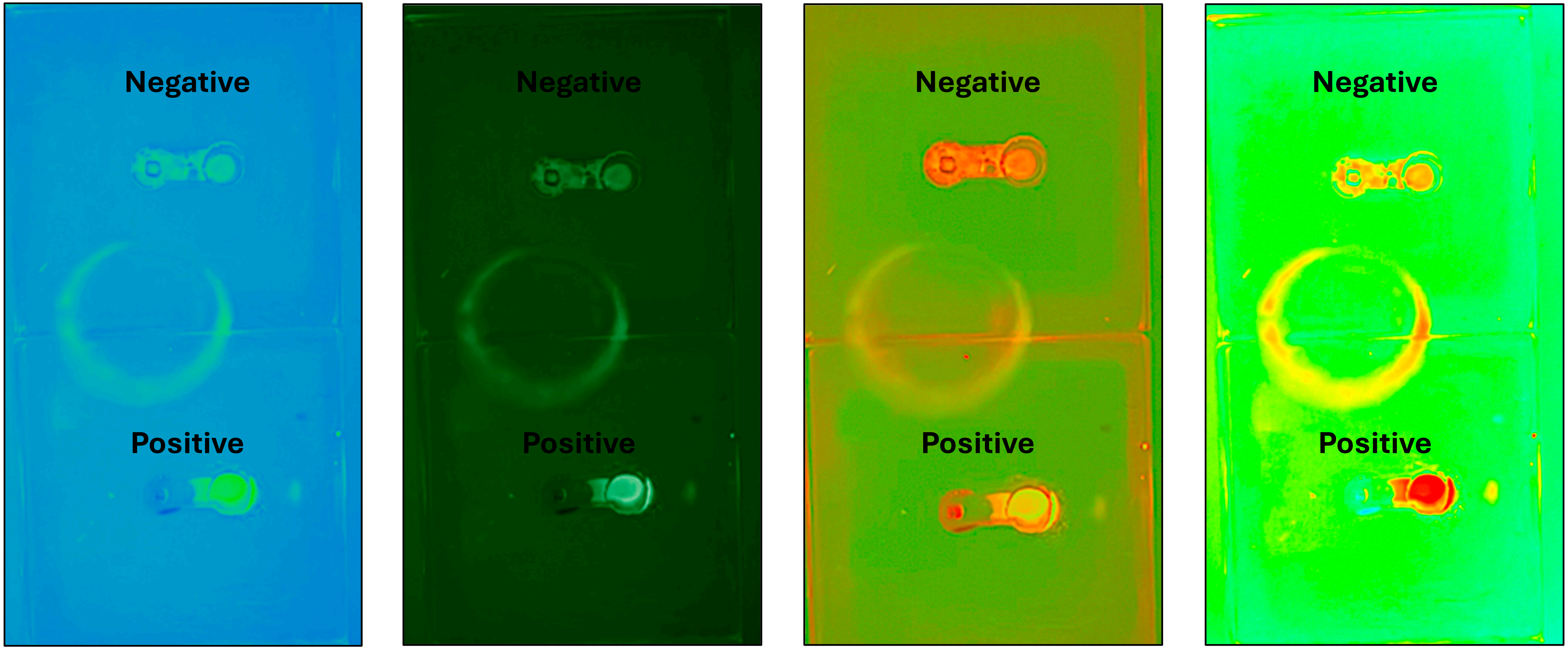

Figure S3: Image analysis of the chip with HDA reactions was generated using ImageJ software, processing data specifically from the blue channel

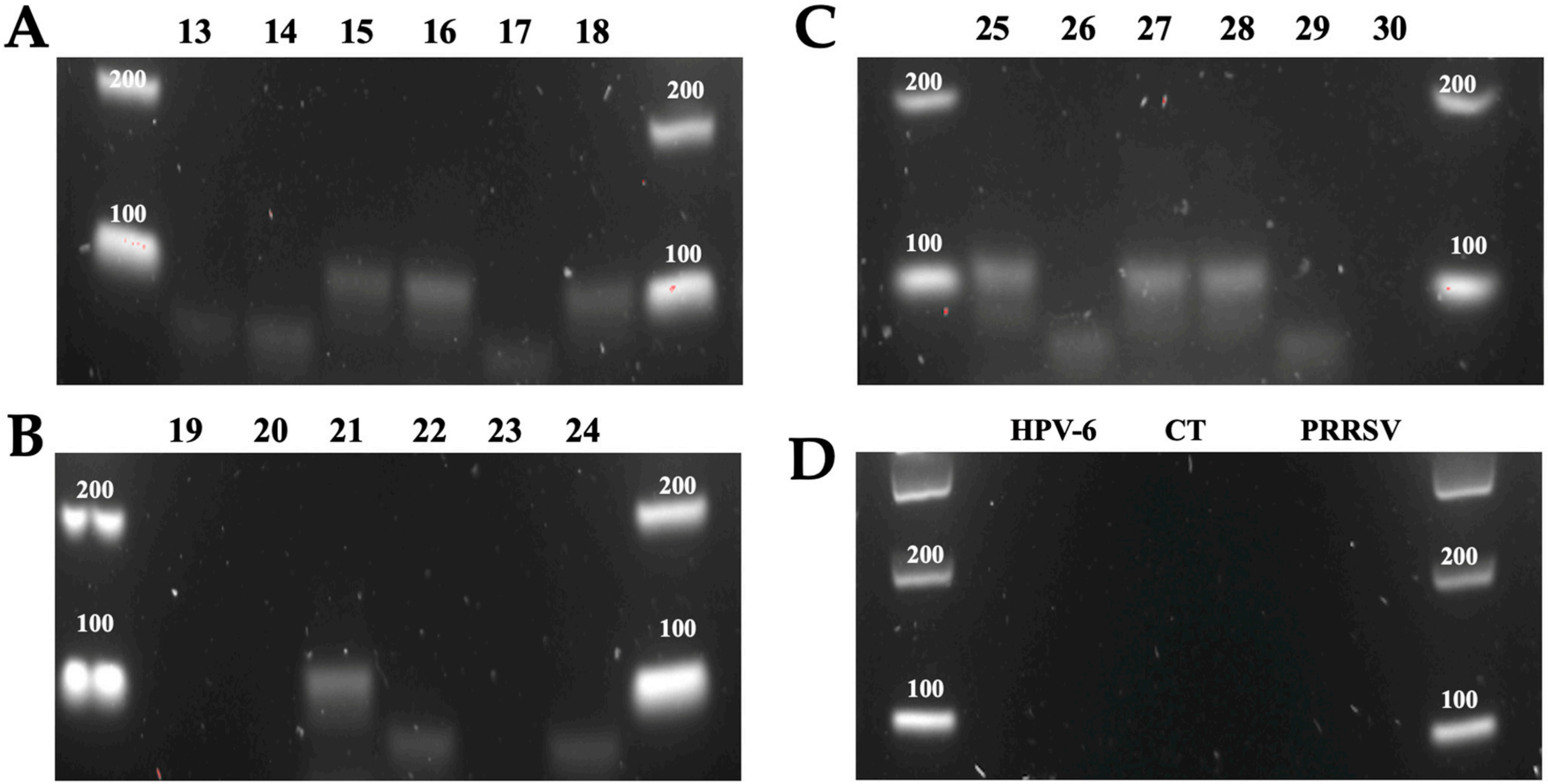

Figure S4. Results of agarose gel electrophoresis from the HDA assay. **A–C)** Results of samples 13 to 30. **D)** Results of negative controls including HPV-6, *Chlamydia trachomatis* (CT), and PRRSV.
